# Using Genomes and Evolutionary Analyses to Screen for Host-Specificity and Positive Selection in the Plant Pathogen Xylella fastidiosa

**DOI:** 10.1128/aem.01220-22

**Published:** 2022-09-12

**Authors:** Tiffany N. Batarseh, Abraham Morales-Cruz, Brian Ingel, M. Caroline Roper, Brandon S. Gaut

**Affiliations:** a Department of Ecology and Evolutionary Biology, UC Irvine, Irvine, California, USA; b Department of Plant Pathology, UC Riverside, Riverside, California, USA; University of Tennessee at Knoxville

**Keywords:** evolution, virulence, genomics, phylogenetic analysis, positive selection

## Abstract

Xylella fastidiosa infects several economically important crops in the Americas, and it also recently emerged in Europe. Here, using a set of *Xylella* genomes reflective of the genus-wide diversity, we performed a pan-genome analysis based on both core and accessory genes for two purposes: (i) to test associations between genetic divergence and plant host species and (ii) to identify positively selected genes that are potentially involved in arms-race dynamics. For the former, tests yielded significant evidence for the specialization of X. fastidiosa to plant host species. This observation contributes to a growing literature suggesting that the phylogenetic history of X. fastidiosa lineages affects the host range. For the latter, our analyses uncovered evidence of positive selection across codons for 5.3% (67 of 1,257) of the core genes and 5.4% (201 of 3,691) of the accessory genes. These genes are candidates to encode interacting factors with plant and insect hosts. Most of these genes had unknown functions, but we did identify some tractable candidates, including *nagZ_2*, which encodes a beta-glucosidase that is important for Neisseria gonorrhoeae biofilm formation; *cya*, which modulates gene expression in pathogenic bacteria, and *barA*, a membrane associated histidine kinase that has roles in cell division, metabolism, and pili formation.

**IMPORTANCE** Xylella fastidiosa causes devasting diseases to several critical crops. Because X. fastidiosa colonizes and infects many plant species, it is important to understand whether the genome of X. fastidiosa has genetic determinants that underlie specialization to specific host plants. We analyzed genome sequences of X. fastidiosa to investigate evolutionary relationships and to test for evidence of positive selection on specific genes. We found a significant signal between genome diversity and host plants, consistent with bacterial specialization to specific plant hosts. By screening for positive selection, we identified both core and accessory genes that may affect pathogenicity, including genes involved in biofilm formation.

## INTRODUCTION

Bacteria exhibit extensive, intraspecific variation in genome content. This variation is the raw material for evolutionary adaptation, including the evolution of pathogenicity and virulence (1–4). One example of genome variation comes from an early study of Escherichia coli that compared two pathogenic strains and one nonpathogenic laboratory strain (5). Of the entire set of protein coding genes annotated by the three genomes, only 39.2% were shared among the three isolates. Intriguingly, the two pathogenic strains each had 1,300 unique genes, while the laboratory strain had only 585, suggesting that genes that vary across accessions (i.e., accessory genes) contribute to virulence. Similar patterns have been illustrated for plant pathogens (6, 7). In *Xanthomonas*, for example, horizontal gene transfer (HGT) has shuffled virulent accessory genes from pathogenic strains to previously nonpathogenic strains (4), facilitating the infection of common bean (Phaseolus vulgaris L.). In short, accessory genes contribute to host-pathogen interactions, making them a critical focus for comparative analyses of genome evolution and function.

Here, we investigate variation in the genome content of another plant pathogen. Xylella fastidiosa is endemic to the Americas and was first identified as the causal agent of Pierce’s Disease (PD), an economically devastating disease in grapevines (Vitis vinifera subsp. *vinifera*) (8, 9). *X fastidiosa* causes additional economically and ecologically impactful diseases, such as citrus variegated chlorosis, coffee leaf scorch, oak leaf scorch, and elm leaf scorch, among others. Historically, the geographic distribution of X. fastidiosa was limited to the Americas, but it was recently introduced to the European continent via anthropogenic transmission, which has further expanded its host range and has led to emerging diseases, such as olive quick decline syndrome (OQDS) in Italy (10, 11). X. fastidiosa has since been detected in various plant species across locations in Europe, including France, Spain, and Portugal (12, 13). In susceptible hosts, X. fastidiosa can lead to significant crop losses, and it continues to threaten crops globally (14, 15).

For each of these diseases, X. fastidiosa is transmitted by xylem-feeding insect vectors into the plant host, where it then utilizes cell wall degrading enzymes to systemically colonize the xylem. In the xylem, it forms biofilms that are thought to be integral to pathogenicity (16, 17). Colonization is also governed, in part, by virulence and pathogenicity factors that influence a wide range of bacterial functions (e.g., biofilm formation, host cell wall degradation, regulatory systems, stress responses, and bacterial membrane composition), although it is likely that other abiotic factors (such as plant drought stress) also contribute to disease progression (13). Given its economic impact, the effects and mechanisms of X. fastidiosa infection have been studied widely, especially in grapevines (18). However, many pathogenicity factors likely remain undiscovered, and crucial questions remain unanswered regarding the genetic factors that govern host-pathogen interactions and potential host specialization (13).

In this context, it is helpful to recognize that X. fastidiosa consists of three commonly recognized subspecies that form distinct phylogenetic clades: subsp. *fastidiosa*, *multiplex*, and *pauca.* Each subspecies has unique phenotypic characteristics and DNA markers (19). Two other subspecies, *morus* and *sandyi*, have also been suggested, though they are not recognized as broadly (9). In fact, *morus* is believed to be a product of a recombination event between *fastidiosa* and *multiplex* isolates (8). The recognition of subspecies is critical because initial work suggests that subspecies correlate with specific plant hosts (20). While it has long been known that genetic differences among strains facilitate host-plant specialization (18, 21–23), there is not a clear one-to-one correspondence between pathogen and host. For example, some strains can infect more than one host species, as demonstrated by a strain that causes PD in grapevines and also causes leaf scorch in almonds (21). Consequently, the questions of the evolution and determinants of host specificity are still central in understanding the distribution and effects of this pathogen.

In this study, we analyze the genome evolution of X. fastidiosa among isolates from different plant hosts. Our study is not unique in some respects, as numerous comparative genomic studies of X. fastidiosa have been published already. Many of these studies have focused on clarifying phylogenetic relationships. For example, Marcelletti and Scortichini (2016) (19) studied 21 genomes to resolve taxonomic relationships among subspecies, Giampetruzzi et al. (2017) (24) extended sampling to 27 genomes, in part to place a novel strain (ST53) in the broader X. fastidiosa phylogeny, and Denancé et al. (2019) (25) used kmers from 46 genomes to untangle species and subspecies relationships. Another recent study compared the X. fastidiosa populations from Central/South America (Costa Rica, Brazil), North America (California, Southeastern United States), Europe (Spain, Italy), and Asia (Taiwan) to elucidate the evolutionary origins of the subsp. *fastidiosa* and *pauca* (26). Still, other studies have focused on populations. For example, Vanhove et al. (2020) isolated and sequenced X. fastidiosa subsp. *fastidiosa* from symptomatic grapevines from five different California locations (27).

One common theme of genomic studies is that they identify the set of genes that are present in most samples (i.e., core genes) and use those genes as the basis upon which to perform phylogenetic inference. These phylogenies have been used for various purposes. For example, two recent papers have used phylogenies to explore the question of host specificity. In one, Uceda-Campos et al. (2022) found that X. fastidiosa isolates grouped on the phylogeny by geography but not by plant host species, suggesting that host specificity is not correlated with phylogenetic relationships or genetic divergence (28). In contrast, Kahn and Almeida (2022) used the phylogeny to infer the ancestral character states of plant hosts and found that the ancestral host plant could be inferred for most ancestral nodes (29). They concluded that genetic history affects the host range and also identified ~30 genes whose presence or absence correlated with specific plant hosts.

In this study, we combined 20 new X. fastidiosa genomes with publicly available data to build a data set for a molecular evolutionary analysis and to investigate patterns of host specificity in a phylogenetic context. For the host-specificity analyses, we focused on core genes, but we also assessed the phylogenetic signal, patterns of gene gain and loss, and potential host associations of accessory (i.e., noncore) genes. Our goals for these analyses were to add to the growing literature about genetic correlations between phylogenetic history and host specificity and also to further consider the dynamic evolution of accessory genes in this context (29). In addition, we performed extensive analyses of the ratio of nonsynonymous to synonymous (dN/dS or *ω*) substitutions to identify genes under positive selection (*ω* > 1.0). Genes under positive selection may be involved in arms-race (or Red Queen) dynamics between pathogens and hosts (30, 31). In other systems, *ω* analyses have identified genes with functions that contribute to host defense, and they have also discovered entirely new sets of genes and pathways involved in pathogen-host interactions (32–34). Here, we apply tests for positive selection in the hope of gaining insight into the sets of genes that may affect host-pathogen interactions.

## RESULTS

### Core and accessory genes in *Xylella*.

**FIG 1:**
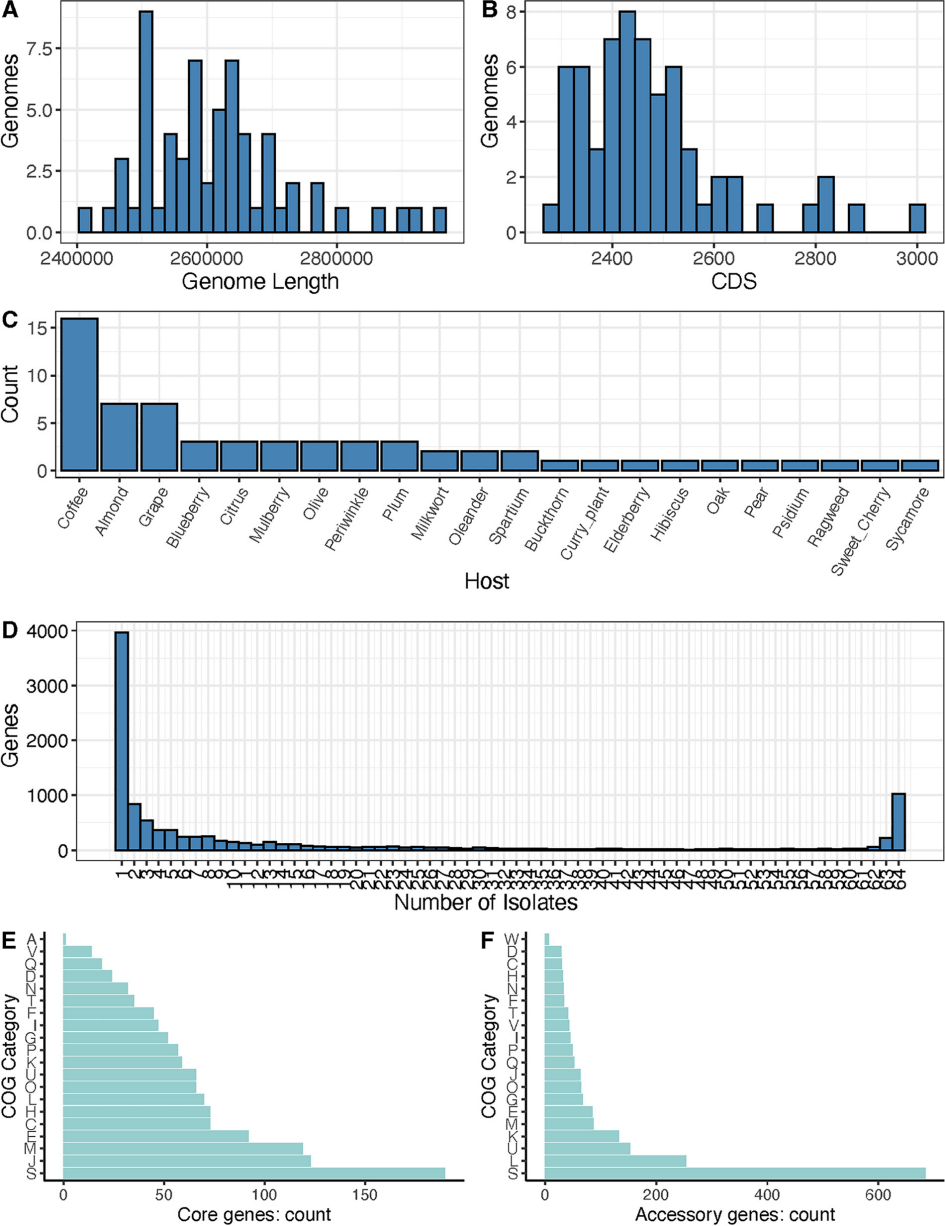
Histograms reporting the characteristics of the 64 *Xylella* genomes. (A) Genome lengths, exhibited in base pairs. (B) The number of genes within the genomes. (C) A histogram of the plant species from which the genomes were isolated. (D) A histogram of the number of genes found in *x* number of genomes. This histogram shows, for example, that nearly 4,000 genes were found in only of one the genomes out of the entire sample of 64 genomes, whereas 1,024 genes were found in all 64 genomes. (E) The distribution of functional categories for the set of 1,257 core genes. (F) The distribution of functional categories for the set of 9,220 accessory genes. A key to the COG categories for panels (E) and (F) is in Fig. S4.

To investigate genome evolution in X. fastidiosa, we sequenced 20 novel X. fastidiosa genomes using hybrid approaches and retrieved publicly available genomes and raw sequencing data (Tables S1 and S2). After filtering for the isolation source and the genetic distance, we retained a sample of 63 genomes that were broadly distributed among the subspecies. All of our analyses were performed on this final set of 63 X. fastidiosa genomes with the X. taiwanensis outgroup. The X. fastidiosa genomes ranged in size from 2.42 Mb to 2.96 Mb, with an average length of 2.61 Mb (Fig. 1A) and an average of 2,478 predicted coding sequences (CDS) (Fig. 1B). The samples were extracted from 22 plant hosts that represented 12 botanical orders (Fig. 1C).

We categorized each gene as either core (present in 95% or more of the X. fastidiosa samples) or accessory (35). Across all 64 genomes, we identified 10,477 genes within the pan-genome. Of those, 1,257 were core genes, and 9,220 were accessory genes, with nearly 4,000 genes found in only a single isolate (Table S4; Fig. 1D). We performed functional analyses on both the core and the accessory gene sets by grouping protein coding sequences into clusters of orthologous genes (COG) (Fig. 1E and F). We compared COG category rankings between the core and accessory gene sets, and a statistically significant difference was found (paired Wilcoxon rank sum test; *P* = 0.0001). After excluding genes with unknown functions, the largest COG categories in the core gene set were “translation, ribosomal structure, and biogenesis” (123 genes), “cell wall/membrane/envelope biogenesis” (119 genes), and “amino acid transport and metabolism” (92 genes). In contrast, the largest categories for accessory genes were “replication, recombination and repair” (547 genes), “intracellular trafficking, secretion, and vesicular transport” (364 genes), and “transcription” (298 genes). Additionally, we investigated the core and accessory gene lists for significant Gene Ontology (GO) based enrichment of specific biological processes (Tables S5 and S6).

### Phylogenetic patterns of core genes, accessory genes, and hosts.

**FIG 2:**
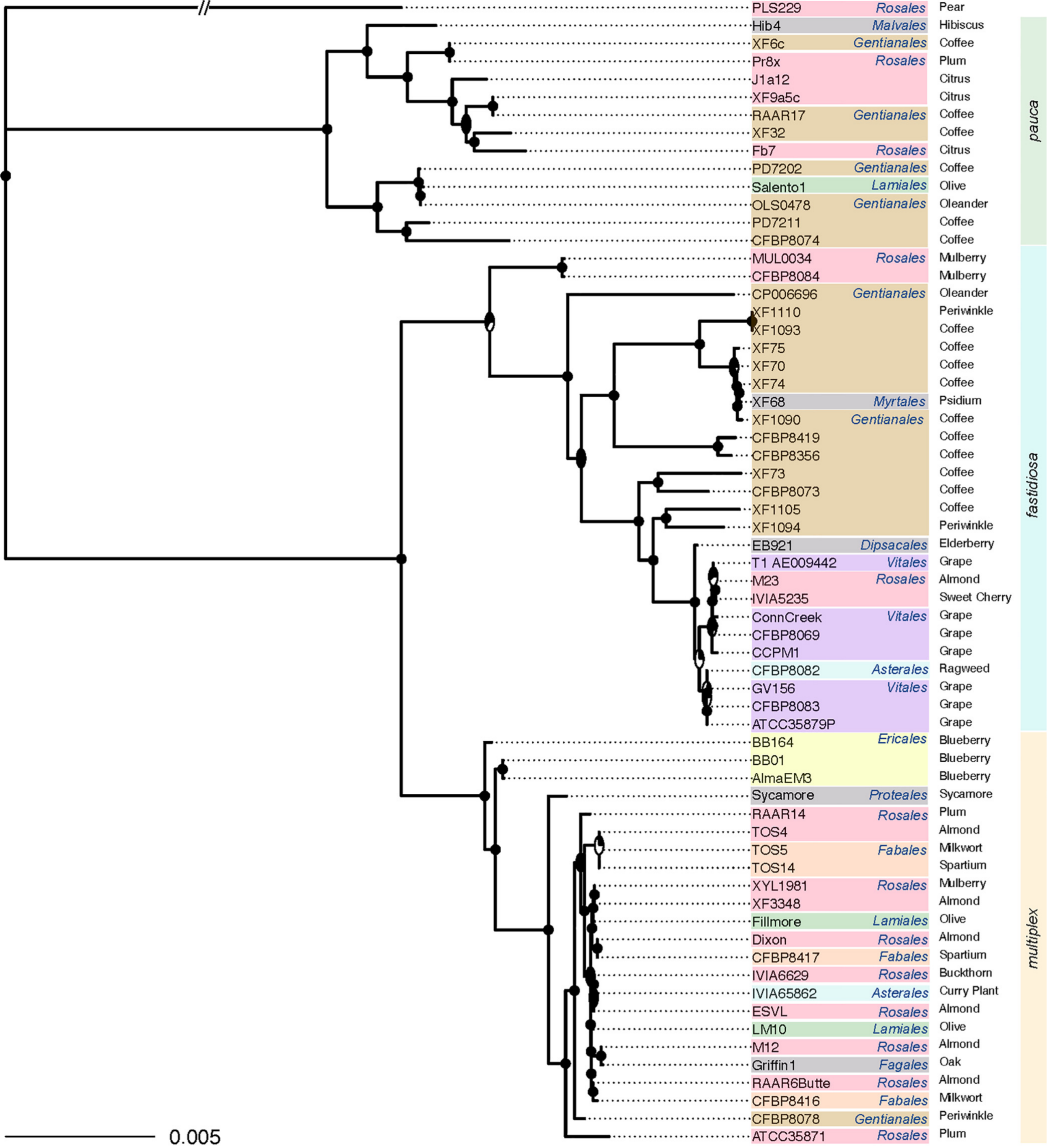
The inferred phylogeny of the 64 *Xylella* genomes, based on maximum likelihood inference on the core gene alignments. Each isolate is labeled at the tips and is colored according to the order of the plant isolation source (host). The common name of the host is provided to the right of the order information. The three X. fastidiosa subspecies are indicated, as are the bootstrap values at each node. The bootstrap values are pie charts, where black represents the percentage of bootstrap support. The scale bar reflects the magnitude of sequence divergence per nucleotide site.

To explore phylogenetic relationships, we constructed a maximum likelihood phylogeny based on a subset of 1,024 genes that were present in all 64 isolates. The topology was highly supported, displaying a mean bootstrap support of 93.75% across all nodes, with a median of 100% (Fig. 2). The lowest bootstrap supports were primarily found at nodes separating the X. fastidiosa strains that were isolated predominantly from grapevines, reflecting relatively low evolutionary divergence among these samples. As expected (25), isolates clustered into three distinct clades representing the three main subspecies (ssps. *fastidiosa*, *multiplex*, and *pauca*), with 27, 23, and 13 isolates in each clade, respectively. To account for the possibility that homologous recombination impacted the resolution of the core phylogeny, we extracted regions of the core gene alignment that had an apparent history of recombination (36), ultimately removing 85.1% of the alignment. The phylogeny inferred from this alignment was nonetheless highly congruent with the phylogeny that did not consider recombination. Only five accessions had altered positions between the recombination-adjusted and nonadjusted trees (Fig. S3).

**FIG 3:**
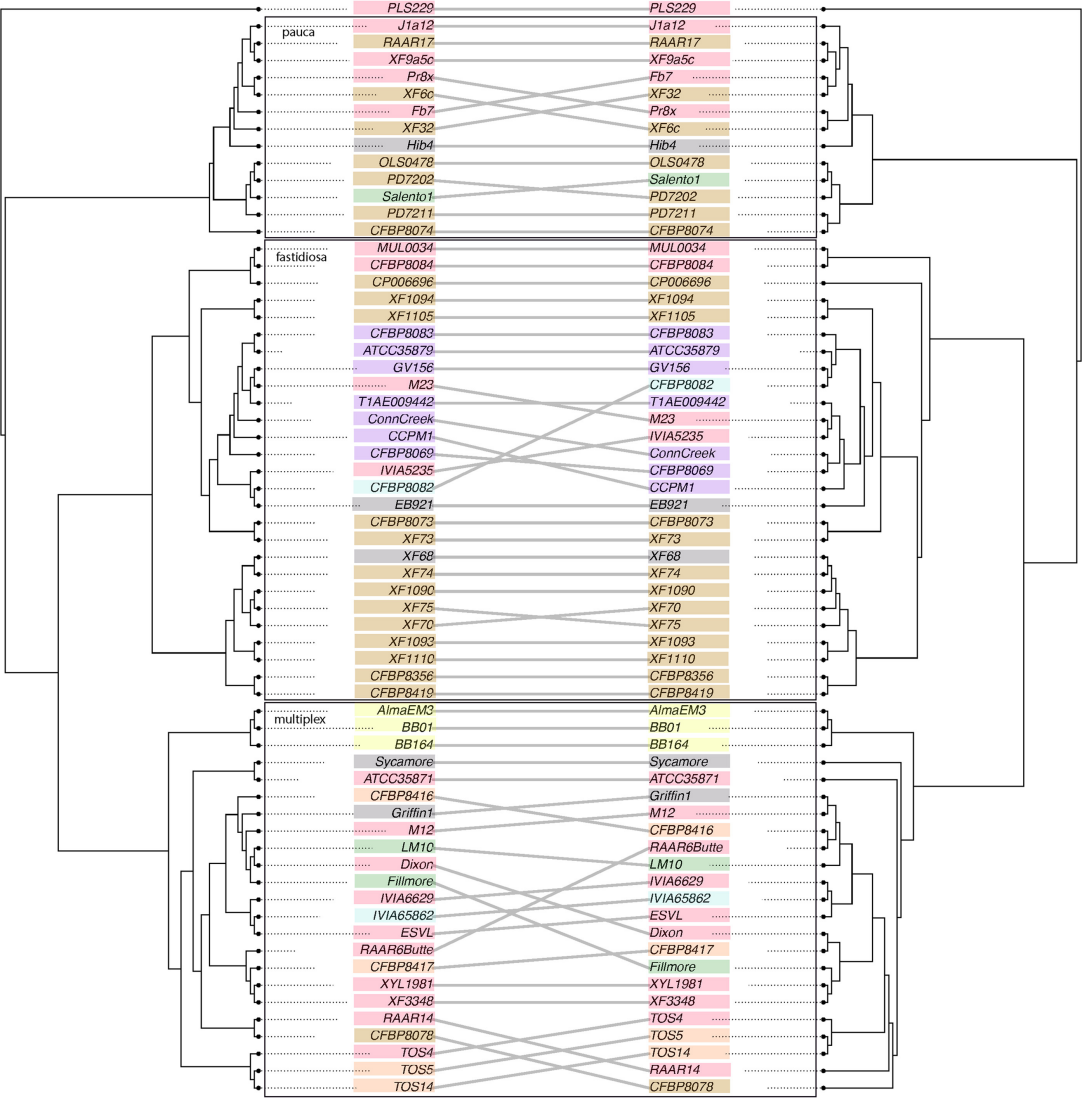
A comparison of a neighbor-joining (NJ) tree, which is based on distances due to gene presence or absence (on the left), to the likelihood tree, which is based on the core gene alignments (from Fig. 2, on the right). As in Fig. 2, the isolates are labeled at the tips of the trees, with the colors representing plant order. Both phylogenies contain three main X. fastidiosa clades, representing the three subspecies. Lines connect the same isolate between the two trees, with angled lines representing topological discordance between phylogenies. The three *Xylella* subspecies are outlined with a black box and are labeled.

To investigate the general evolutionary patterns of the accessory gene complement, we compared the core gene phylogeny against a phylogeny based on accessory gene composition (Fig. 3). Both the core gene and the accessory gene phylogenies clustered into three groups, and all members of the groups were consistent between phylogenetic treatments. This pattern broadly suggests that accessory genes, while defined by their inconstancy, are not exchanged *en masse* to a large enough extent to alter phylogenetic signals among subspecies. Within subspecies, however, relationships at the tips of the phylogeny often differed between the core and accessory trees. As an example, the cluster corresponding to *multiplex* displayed the most discordance between the core and accessory trees, with all operational taxonomic units (OTUs) contributing to phylogenetic incongruence (Fig. 3). Interestingly, our *multiplex* sample also had more plant host species than did our *fastidiosa* and *pauca* samples, suggesting the possibility (but by no means proving) that host factors may affect or moderate genome content (29). Nonetheless, we found a significant correlation between the distance matrices based on the core and accessory phylogenies (Mantel test; *R* = 0.1144, *P* = 0.019), which is consistent with the fact that the two trees have the same three major clades. The overarching impression of these analyses is that accessory gene composition does not turn over so rapidly, due to HGT or other mechanisms, to erase the phylogenetic and historical signals of subspecies diversification within X. fastidiosa.

We used both species phylogenies (based on alignments with and without putative recombinant regions) to test for associations between the X. fastidiosa samples and their isolation sources (i.e., geographic location or host plant information) using an analysis of similarities (ANOSIM, see Materials and Methods). There was a weakly significant phylogenetic association (ANOSIM; *R* = 0.08178, *P* = 0.042) between the geographic location and the phylogeny built from the full core gene alignment (ANOSIM; *R* = 0.08178, *P* = 0.042) but not with the phylogeny built from the nonrecombinant regions (ANOSIM; R = −0.004147, *P* = 0.4895). Applying the same approach to the host species revealed a significant phylogenetic signal for both phylogenies (ANOSIM; *R* = 0.1381, *P* = 0.047; for the nonrecombining regions only, ANOSIM; *R* = 0.6698, *P* < 1×10^−4^). Since X. fastidiosa infects a wide range of plants, we also retrieved the taxonomic order of each plant host to test for a phylogenetic signal at a deeper taxonomic level, recapitulating the significant association with both phylogenies (ANOSIM; *R* = 0.3152, *P* < 0.0001; for the nonrecombining regions only, ANOSIM; *R* = 0.1226, *P* = 0.0198). In other words, strains isolated from plants within the same taxonomic order were more phylogenetically similar to one another than were isolates taken from unrelated plants.

We hypothesized that accessory genes are crucial in pathogen-host interactions. Therefore, we repeated the ANOSIM analyses with a distance matrix based on the presence and absence of accessory genes (Fig. 3). We found a significant association between accessory gene content and geographic isolation source (ANOSIM; *R* = 0.4553, *P* = 0.5307) and a weakly significant association between accessory gene content and host species (ANOSIM; *R* = 0.1503, *P* = 0.0372). The association was lost, however, at the level of plant order (ANOSIM; *R* = 0.02367, *P* = 0.3033). Overall, associations were less evident based on accessory gene content versus the core-gene phylogeny.

**FIG 4:**
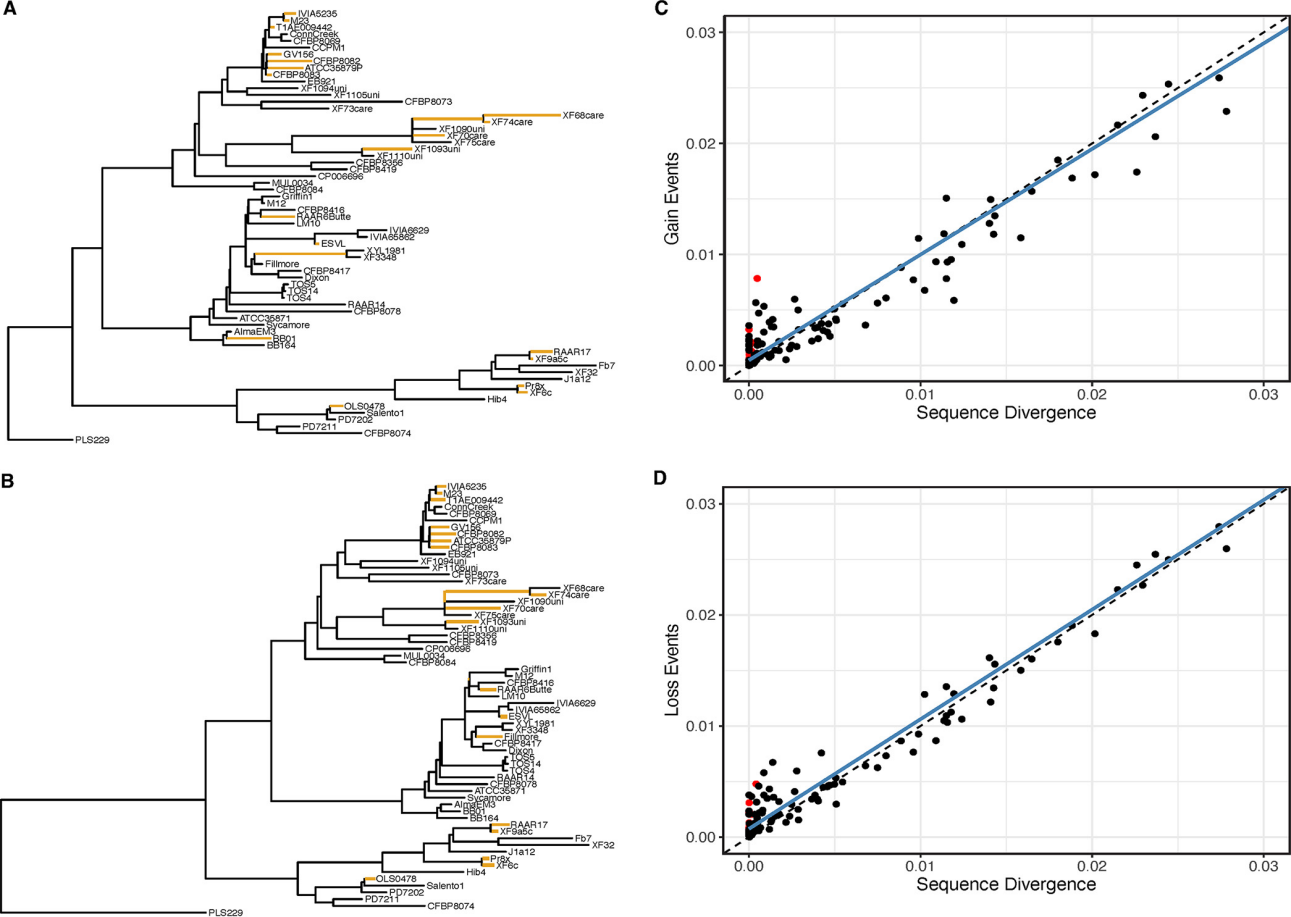
The results of gene gain and loss analyses. (A) The phylogeny of the isolates, with branch lengths proportional to the number of gene gain events. The colored branches are branches with outlier gene gain rates. (B) The phylogeny of the isolates, with branch lengths proportional to the number of gene loss events. The colored branches are branches with outlier gene loss rates. (C) A plot of the gene gains against sequence divergence. In the plot, each dot represents one of the 125 branches of the phylogeny. Outlier dots are colored red. (D) As in panel C, with gene the losses plotted against sequence divergence.

Concerning gene gain and loss, the sheer number of accessory genes indicates that the genome content of X. fastidiosa is, like those of other microbes (37, 38), shaped by extensive gene gain and loss events that are probably mediated by HGT (39). We were interested in assessing the pattern of gene gain and loss across the phylogenetic tree, hypothesizing that both could be enhanced on branches that lead to host shifts. We used GLOOME to estimate the number of gains and losses of accessory genes across the X. fastidiosa phylogeny and represented those estimates phylogenetically (Fig. 4). Ignoring the branch leading to the *X. taiwanensis* outgroup (PLS229), the internal branches discriminating the X. fastidiosa subspecies were estimated to average ~550 separate gene gain and gene loss events. The remainder of the tip and ingroup branches averaged ~100 gene gain and loss events (average gains/branch = 92.8 genes; average losses/branch = 100.0 genes; Fig. 4A and B).

While it is useful to estimate the number of gains and losses on each branch, we thought it more helpful to normalize the numbers of estimated gain and loss events by the branch lengths, which were estimated from the sequence analysis of the core genes. This normalization by branch length converted the number of gene gains and losses to rates of gene gain (or loss), relative to the sequence divergence. We then sought to identify branches with aberrantly high rates of gene gain or loss (Fig. 4C and D), which would be indicative of branches with especially notable turnover of accessory genes. As was found in a previous microbial study (40), we found that most of the phylogenetic lineages with outlier rates were located at the tips of the phylogenetic tree. For example, of the 21 branches with high rates of gene gain, 19 were at the tips of the phylogeny (Fig. 4A). Similarly, 18 of the 21 branches with high rates of gene loss were external branches. These observations suggest features about the evolutionary dynamics of the genetic turnover (see Discussion).

### Characterizing selection with *ω*.

We characterized selection on individual genes by estimating the dN/dS ratio (*ω*). We especially sought to identify genes that experienced positive selection (i.e., *ω* > 1.0), as these could indicate a potential signal of genes that contribute to dynamics between the pathogen and its hosts. To do so, we applied a series of nucleotide substitution models to individual genes, ultimately resulting in tests for positive selection on two levels: globally across a phylogeny and across codon sites (see Materials and Methods). For these tests, we examined the full complement of 1,257 core genes, a subset of 3,691 accessory genes, and a set of 187 multicopy genes.

**FIG 5:**
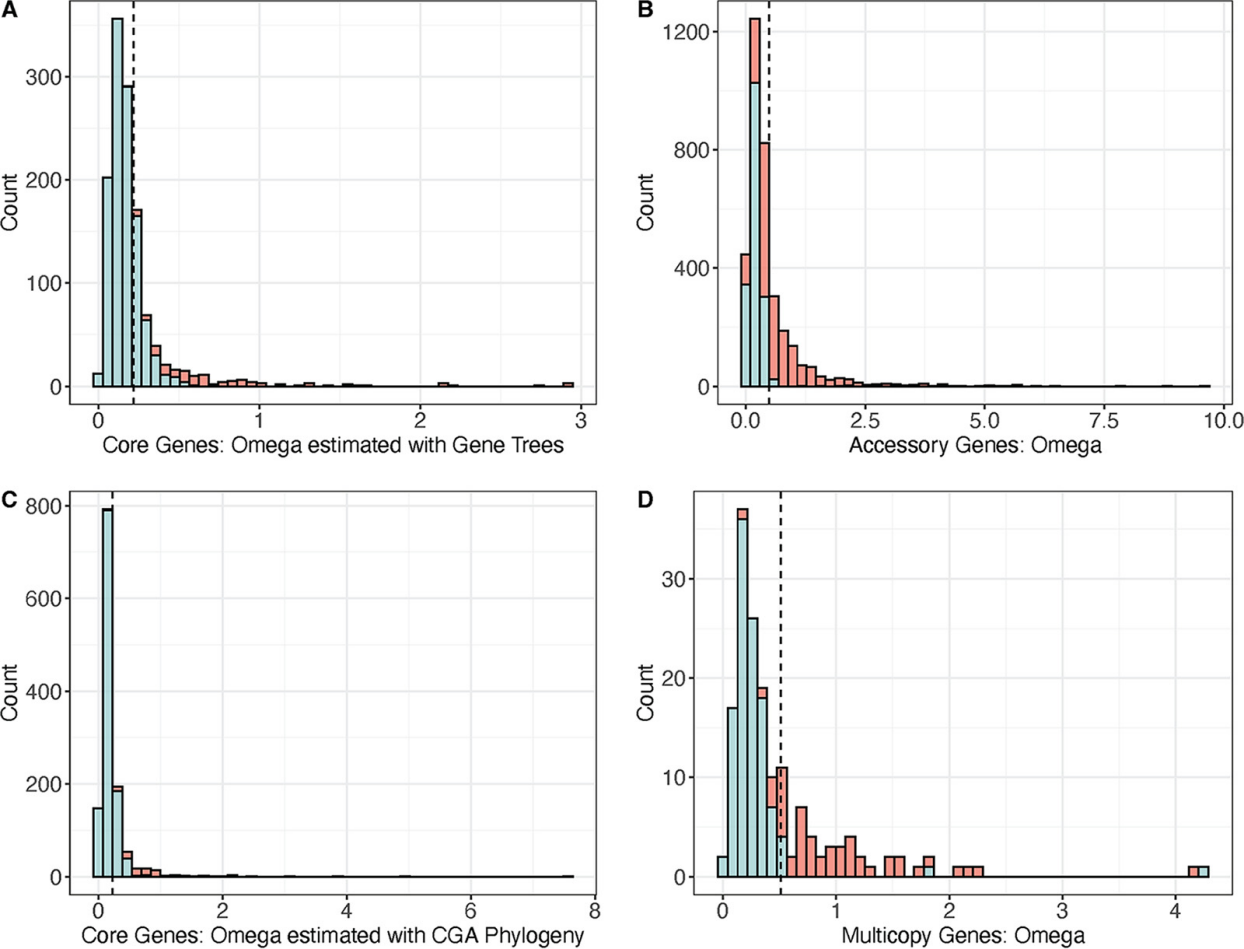
Estimated values of ω under M0 (the one-ratio model) in the core and accessory genes. The distribution of the $\hat{\omega }$ values is plotted for (A) the core genes estimated with gene trees, (B) the accessory genes with gene trees, (C) the core genes estimated with the core gene alignment (CGA) phylogeny, and (D) the multicopy genes with gene trees. The histogram bars are shaded to reflect the outcome of the likelihood ratio test (insignificant tests are colored red, and significant tests are colored blue) between a model that estimated $\hat{\omega }$ and a model with ω fixed to 1.0. The horizontal dashed line denotes $\hat{\omega }$ for each gene set.

Concerning testing selection globally for each gene, we first estimated a single *ω* value for each gene, using a method that assumes that *ω* is constant across all branches of the entire gene tree and across all codons in the nucleotide alignment. Applied to the core genes, *ω* estimates ($\hat{\omega }$), ranged from 0.01048 to 2.92803 with an average of 0.21973 (Fig. 5A). Nineteen core genes had $\hat{\omega }$ values higher than 1.0, but none of these were significantly >1.0 (*P* > 0.01, FDR correction). In fact, the vast majority (1,144 of 1,257) of the core genes had $\hat{\omega }$ significantly <1.0 (*P* < 0.01, FDR correction; Fig. 5A), reflecting pervasive purifying selection. The range of $\hat{\omega }$ was substantially broader for the accessory genes, ranging from $\hat{\omega }$ = 0.0001 to 9.60069, with an average of 0.51443 (Fig. 5B). Among the accessory genes, 367 (9.9%) had a global estimate of *ω* > 1.0, but only eight displayed statistically significant evidence for positive selection. These eight genes were candidates to encode proteins involved in host-pathogen interactions, but seven of the eight were annotated as hypothetical genes (Table S7). Overall, the average $\hat{\omega }$ was significantly higher in the accessory gene set compared to the core genes (Welch’s *t* test; *P* < 2.2×10^−16^), reflecting either lower purifying selection against these genes, more positive selection, or both.

We also identified 187 genes that had 2 or more copies within a single accession in a syntenic context but that were single copy in other accessions. We performed a *codeml* analysis to estimate *ω* for each multicopy gene, and $\hat{\omega }$ ranged from 0.02272 to 4.26800, with an average of 0.51129 (Fig. 5D). Over half of the genes had $\hat{\omega }$ significantly <1.0 (59.4%, *P* < 0.01, FDR correction), and only one, a hypothetical gene (*group_1109*), had $\hat{\omega }$ significantly higher than 1.0 ($\hat{\omega }$ = 1.85845, *P* < 0.01, FDR correction).

The global test is a conservative criterion by which to search for positive selection. Accordingly, we turned to an alternative method that tests for variation in *ω* among codon sites and identifies whether sites are under positive selection. To do so, we ran sites models in *codeml*, representing a group of nested models. For completeness, we first compared sites model M0, which represents the null hypothesis that there is a single *ω* value for all sites, against sites model M3, which permits *ω* to vary among sites. In the core genes, the likelihood ratio test was significant for 501 genes (*P* < 0.01, FDR correction). We then took this set to compare and test for positive selection using the sites models. A total of 67 core genes had evidence of positive selection among sites (*P* < 0.01, FDR correction). We also tested for positive selection on codon sets within the 3,691 accessory genes, using the same approach. Of the total, 895 displayed evidence of a variable *ω* value among sites (*P* < 0.01, FDR correction), and 201 yielded evidence of positive selection (*P* < 0.01, FDR correction). Finally, we applied the sites models to the set of 187 multicopy genes, yielding another 33 genes with evidence of positive selection. In summary, 5.3% (i.e., 67 of 1,257) of the core genes, 5.4% (201 of 3,691) of the accessory genes, and 17.6% of the multicopy genes had significant evidence of at least one codon with an apparent history of positive selection. Among the 201 accessory genes, four (*cya*, *group_454*, *group_1057*, and *group_3542*) also displayed evidence for positive selection via the global test.

## DISCUSSION

Host-pathogen interactions can drive the rapid evolution of pathogenic bacteria, particularly for genes involved in arms-race dynamics (30, 41). Here, we investigated the genomic evolution of the plant pathogen, X. fastidiosa, through a comparative genomic analysis of genomes representative of the diversity across the species, based on a sample set of 64 genomes. The sample was isolated from 23 different plant hosts (Fig. 1C) from throughout the world (Fig. S1). With these data, we constructed a pan-genome that contained 1,257 core genes and 9,220 accessory genes, similar to those of previous studies (24, 42). Of the core genes, the majority were, as expected (43), involved in essential cellular processes, such as translation, cell wall biogenesis, and amino acid metabolism (Fig. 1E). We used the set of core genes to infer a maximum likelihood phylogeny, either with or without adjusting for the putatively recombining regions of the genome (Fig. 2; Fig. S3). As with the previous systematic treatments of X. fastidiosa (19, 25, 44), both phylogenies identified three clades corresponding to the three main subspecies (*fastidiosa*, *multiplex*, and *pauca*).

We employed both phylogenies to investigate the relationship between the X. fastidiosa phylogeny and the plant host. The question of host specialization was first addressed using phylogenetic approaches with multilocus sequencing typing (MLST) data. In this work, Sicard et al. (2018) (8) generated MLST data from 7 housekeeping genes from 50 X. fastidiosa genotypes. After building a phylogeny, they tested coevolutionary relationships between the host species and the X. fastidiosa MLST types but found no significant evidence of coevolution, implying a lack of host specialization. This topic was recently revisited with full genome data (28, 29), but the results were inconsistent between studies. Uceda-Campos et al. (2022) (28) found no evidence that the plant host species clustered on their X. fastidiosa phylogeny, but the samples did cluster by geography. In contrast, Kahn and Almeida (2022) (29) inferred the ancestral character states of plant hosts on the X. fastidiosa phylogeny and were able to resolve the character states of some deep nodes. They inferred, for example, that coffee plants were the ancestral host species for the node separating X. fastidiosa subsp. *fastidiosa* from other subspecies. These patterns suggest that phylogenetic history is associated with specific plant hosts and host ranges.

The disagreement among previous studies, and the fact that all such analyses are properties of the sampled isolates, makes this issue worthy of further assessment. In our study, we found a significant, nonrandom association between phylogenetic relationships and both the species and taxonomic order of plant hosts (*P* < 0.0001) based on core phylogenies. These results are consistent with some level of specialization of X. fastidiosa to plant hosts and with the results of the recent analysis by Kahn and Almeida (2022) (29). Moreover, these results were robust to phylogenetic - that is, the inclusion or exclusion of genomic regions were inferred to have histories of recombination. Although it is difficult to quantitatively compare ANOSIM results across studies, it is worth noting that the association of X. fastidiosa to plant order is similar in magnitude to the association between a gut colonizing bacterium (*Bifidobacterium*) and the host species from which it was isolated (45).

Given some evidence for host specialization, we hypothesized that it is driven in part by accessory gene content. Under this hypothesis, we predicted that an association between genes and hosts should be as (or more) pronounced for the accessory genes as for the core genes. Instead, we found no significant association between the accessory gene complement and taxonomic order and only a weak association with plant species. Our results are unlike, for example, the case of the bifidobacteria, where the association with the host species was nearly as strong for the accessory genes as it was for the host genes (45). We cannot be sure why we do not detect a signal for the host specialization of the accessory genes, but we can think of three explanations. One is that host associations, to the extent they exist, are not driven by accessory genes but by evolutionary divergence in core genes. Another is statistical power; because there are many more sequence changes among the core genes than there are changes in accessory gene content, the distance matrix for the core genes likely has a higher signal-to-noise ratio than does the accessory gene content. Finally, if accessory genes do mediate host shifts, it is possible, and even likely, that only a subset of the accessory genes drive these shifts. Under this scenario, there may be significant associations for a small subset of accessory genes, but the signal of this association is weak across the entire accessory gene set. This conjecture seems reasonable, given that Kahn and Almeida (2002) found that the presence or absence of a subset of only ~30 accessory genes correlated with the plant host. In addition, it is worth emphasizing that X. fastidiosa interacts not only with plants but also with insect vectors and microbial communities, such that some subset of accessory genes likely contributes to these interactions instead of those with plant hosts.

### The pattern of gene gain and loss events.

Another potential tool to study adaptation to specific hosts is by examining shifts in gene composition through gene duplication, deletion, or HGT events (46, 47). We estimated the number of gene loss and gain events along the core gene phylogeny and normalized those numbers relative to the sequence divergence. Using this approach, we found that most branches followed a consistent rate of gene gain or loss relative to the sequence divergence. The fact that the accessory gene phylogeny recapitulates the three subspecies (Fig. 3) suggests, along with previous evidence, that X. fastidiosa evolves predominantly through vertical inheritance and intraspecific recombination rather than through HGT from other bacterial species (20, 48).

We have, however, identified 19 and 18 lineages with enriched gain or loss events, respectively, and most of these branches were at the tips of the phylogeny. Again, a potential explanation for these gain and loss dynamics is that they reflect host shifts. There are some isolated examples that are consistent with this hypothesis. For example, isolates XF6c, Pr8x, RAAR17, and OLS0478 in *pauca* have branches with enriched gene gains (Fig. 4A). Two of these (OLS0478 and Pr8x) were isolated from oleander and plum, respectively, and they are the only isolates associated with those plant hosts in their clades, suggesting a host shift. More globally, however, the evidence for this hypothesis is unconvincing. When we, for example, contrast gene gains between pairs of sister taxa with the same plant host, 3 of the 16 sister pairs had enriched rates of gene gain. This proportion of enriched branches was not significantly lower than that of the remainder of the tree (*P* > 0.05; Fisher’s exact test), despite the fact that the sister taxa did not experience a host shift. All of these inferences are, of course, dependent on our sample and ignore the vector component of the X. fastidiosa life cycle. So, there are limitations to our conclusions. At present, however, the evidence for an association between host shifts and enhanced gene gain and loss events is weak.

This leaves unexplained the pattern of the enriched rates of gene gain and loss at the tips of the tree. We suspect that this pattern is analogous to patterns of mutations in populations, as suggested previously (40). New mutations begin as rare, low frequency variants in single individuals. Eventually, most of these mutations are removed by the processes of genetic drift and natural selection such that there are more new mutations in populations than old mutations. In a phylogenetic context, these new mutations would be evident at the tips of the trees, so it may be reasonable to expect higher effective rates of gene gain and loss in the “newest” phylogenetic branches. This explanation only has credence, however, if the observed gain and loss events are both frequent and recent (i.e., newer than the sequence mutations that define the tip branches).

### The identification of positively selected genes.

Many previous studies have implicated genes and their protein products in ongoing arms-races between pathogens and their hosts (49, 50). One way to approach this question is agnostic to function, which is to screen for genes with a history of positive selection. Ours is not the first attempt to detect selection in X. fastidiosa genomes. Previous studies have searched for selection by comparing levels of polymorphism or rates of synonymous and nonsynonymous mutations in the core genome using Tajima’s D and the McDonald-Kreitman test (26, 42). Other work has measured *ω* in core genes but without statistically testing for positive selection (48) or by applying a global test for *ω* values that are greater than 1.0 (27). To our knowledge, no other study of X. fastidiosa has either tested for positive selection in accessory genes or applied codon sites models. The set of positively selected X. fastidiosa genes represents candidate pathogenicity factors that mediate interactions with the environment, including the plant host, insect vectors, or members of the microbial community.

To study positive selection, we estimated *ω*, which is the ratio of nonsynonymous to synonymous mutations, for each core gene and for each accessory gene found in four or more isolates. In total, this exercise encompassed 5,135 genes: 1,257 core genes, 3,691 accessory genes, and 187 multicopy genes. We began by applying a global test that estimates *ω* over all sites and phylogenetic lineages. This approach can be overly conservative, as a significance test of *ω* > 1.0 requires that positive selection is strong, acts across many sites in a gene, is present in most of the branches of the phylogeny, or all of the above. As expected, we found only a few genes (eight accessory genes in total) that were significant for positive selection with this test. Unfortunately, the annotations of 7 of 8 of these genes yielded few insights into their functions. To explore gene function further, we identified protein domains using the Conserved Domain Database. We found, for example, that the gene *group_7848* contains a VirB3 protein domain, which is part of the Type IV secretory pathway and is commonly associated with the membranes of the bacterial cell. The gene *cya* was also implicated using this test, which encodes adenylate cyclase and plays an essential role in the regulation of cellular metabolism (51). Interestingly, the *cya* protein is involved in the cyclic AMP system, which is a global regulator in Gram-negative bacteria and has been shown to modulate gene expression in pathogenic bacteria (52, 53).

The global test did allow, however, for two broad generalizations about the patterns of selection in X. fastidiosa. First, as a group, the core genes are under strong purifying selection, with most (>90%) having *ω* estimates significantly less than 1.0. Second, accessory genes generally have lower levels of purifying selection, as evidenced by a lower proportion (45%) of significant tests for *ω* < 1.0 and by much higher average $\hat{\omega }$ values (0.21973 versus 0.51443; Fig. 5A). The proportion of significant tests must be compared between genic sets with caution because the smaller sample sizes (*n *= 4 to 59) for the accessory genes likely reduces statistical power, relative to the minimum of 60 samples for all core genes, as do any differences in gene lengths. Nonetheless, the contrasting pattern of *ω* is consistent with the ideas that core genes have conserved biological functions and that accessory genes are more amenable to evolutionary change due to their nonessential, but still potentially biologically relevant, cellular roles (54). Accessory genes may also experience higher variation in their selection dynamics because recombination affects them more than it affects core genes (48).

Given few signals of positive selection with the global test, we turned to codon site models. To our surprise, the proportion of positively selected genes was similar for core genes (5.3%) and for accessory genes (5.4%). The salient question is whether these genes give some clue to function. Of the 67 core genes with evidence for positive selection at the codon level, 40% were unannotated. We performed a functional analysis by grouping the protein coding sequences of these 67 core genes into COG categories to infer cellular functions. Excluding the category of unknown function, the largest category was “cell wall/membrane/envelope biogenesis”, followed by the “amino acid metabolism and transport”, “carbohydrate metabolism and transport”, “translation”, and “intracellular trafficking and secretion” (Fig. S4A).

Of the 201 accessory genes with evidence for positive selection at the codon level, 82% were not annotated for function. The remaining set of 36 genes was enriched for GO categories related to protein secretion by the type IV secretion system (Table S8). To better infer function, we performed a COG analysis and found that the largest categories (excluding the category of unknown function) were “intracellular trafficking and secretion”, “replication, recombination and repair”, and “secondary metabolites biosynthesis, transport and catabolism” (Fig. S4B). Intriguingly, of this set of 201 genes, 50 overlapped with the set of 367 genes that had a gene-wide estimate of $\hat{\omega }$ > 1. While these are especially strong candidates for having a history of positive selection, a disappointing 94% of them were unannotated for function. The three genes with annotations were *cya*, *nagZ_2*, and *bacterial adaptive response A* (*barA*). The gene *nagZ_2* encodes a beta-glucosidase that is important for biofilm formation in Neisseria gonorrhoeae, suggesting that it could play a similar role in X. fastidiosa. It merits further functional analysis, since biofilms are important to the infection cycle (55). *barA* encodes a membrane associated histidine kinase that has a regulatory role in cell division, metabolism, and pili formation, and it has been implicated in regulating the virulence response of uropathogenic E. coli (56, 57). Finally, the multicopy genes also yielded evidence of positive selection, including *cdiA1*, which is part of the secretory contact-dependent growth inhibition (CDI) system that modulates biofilm formation in Acinetobacter baumannii (58).

As a final exercise, we cataloged the incidence of positive selection in a set of 35 genes that have been listed as virulence and pathogenicity factors in X. fastidiosa (13). Of the 35, we could identify 29 in our database based on the PD number annotations and reference sequences (http://www.microbesonline.org/operons/gnc183190.html; Table 1). We expected that this set of 29 genes would be enriched for evidence of positive selection relative to the genomic background because these genes are putatively involved in arms-race interactions. The trend for these genes was in the expected direction, because 4 of 29 (13.9%) were significant versus 301 of 5,135 (5.8%) in the rest of the genome. However, the difference in proportions was not significant (Fisher’s exact test; *P* = 0.1091). Nonetheless, this set of experimental genes is interesting. All four genes with evidence of positive selection encode proteins associated with the membranes of Gram-negative bacteria and are involved in membrane transport or adhesin. Specifically, the genes *fimF*, *xadA*, and *xatA* encode proteins involved in fimbrial adhesion, nonfimbrial adhesion, and biofilm formation, respectively, and the gene PD1311 encodes a protein involved in membrane transport (59–63). Because there is a resolved protein structure for fimF (64), we investigated the location of positively selected codons. Of the four positively selected codons (N80, D87, F137, and D142), one (D87) was in a flexible loop, and a second (D142) comprised part of the second β-sheet of the protein (64). Together this suggests that changes in the amino acid sequence of *fimF* may be impacting its function.

**TABLE 1:** *Codeml* results for experimentally identified virulence and pathogenicity genes, as listed (13)

PD no.	Gene name	Pan-genome classification	No. genomes^*a*^	M0^*b*^	M2a versus M1a *P* value^*c*^
PD0058	*fimF*	Accessory	41	0.31555	**3.25e−08**
PD0062	*fimA*	Accessory	26	0.81255	0.247
PD0233	*rpfB*	Accessory	57	0.16832	1
PD0279	*cgsA*	Core	64	0.14404	1
PD0406	*rpfC*	Accessory	44	0.34502	1
PD0528	*xatA*	Core	64	0.43097	**1.38e-41**
PD0731	*xadA*	Accessory	58	0.39196	**0.004**
PD0732	*xpsE*	Core	64	0.05825	1
PD0814	*wzy*	Accessory	43	0.17675	1
PD0843	*tonB1*	Core	64	0.11374	0.534
PD0848	*pilL*	Core	64	0.18195	1
PD0986		Core	64	0.10828	1
PD1099	*dinJ/relE*	Accessory	25	0.10271	1
PD1100		Accessory	15	0.20708	0.731
PD1284	*algU*	Core	64	0.19261	1
PD1311		Accessory	33	0.42541	**3.47e−05**
PD1380	*csp1*	Core	64	0.15702	1
PD1391	*gumH*	Accessory	46	0.12964	1
PD1394	*gumD*	Core	63	0.11504	1
PD1485	*pglA*	Accessory	59	0.28401	0.114
PD1678	*phoQ*	Core	64	0.1086	1
PD1679	*phoP*	Core	64	0.03272	1
PD1703	*lesA/lipA*	Core	64	0.06614	1
PD1792	*hxfB*	Core	64	0.10828	1
PD1826	*chiA*	Core	64	0.11424	1
PD1856	*engXCA1*	Core	63	0.24034	1
PD1964	*tolC*	Core	64	0.10051	1
PD1984	*gacA*	Core	64	0.13444	1
PD2118	*hxfA*	Core	64	0.10828	1

a The number of genomes, out of 64, in which the gene was detected.

b M0 estimates a single *ω* across the entire phylogeny of sequences.

c The *P* value of the test after FDR correction. Bolded values are significant at *P* < 0.01. The notation e refers to the power of 10.

We must caution that positive selection analyses are subject to false-positives, and they are also dependent on specific analysis features, such as the sample set, the criteria for determining homology, and the sequence alignments. Nonetheless, we have found several genes with some evidence of positive selection that may also contribute to functions that are relevant to infection. We believe that they represent suitable candidates for further functional analyses to elucidate their roles in host-pathogen interactions and perhaps even host specificity.

## MATERIALS AND METHODS

### Novel X. fastidiosa genomes.

Fully extracted DNA from 20 X. fastidiosa isolates were provided by the French Collection of Plant-Associated Bacteria (CIRM-CFBP; http://www6.inra.fr/cirm_eng/CFBP-Plant-Associated-Bacteria) and from the University of California, Riverside. Genomic DNA was prepared for Illumina sequencing using the Illumina Nextera DNA Flex Library Prep Kit, following the manufacturer’s recommendations, and for Pacific Biosciences (PacBio) sequencing with the SMRTbell Express Template Prep Kit 2.0. The SMRTbell libraries had a 10 kb DNA target insert size (Pacific BioSciences, Menlo Park, CA) and used 360 ng of sheared DNA as an input. The DNA libraries were sequenced with both Illumina and PacBio technologies at the University of California, Irvine Genomics High Throughput Facility (https://ghtf.biochem.uci.edu). The Illumina sequencing reads were quality assessed using FastQC, and the reads were trimmed using Trimmomatic v. 0.32 (65, 66), using the default options. The PacBio sequencing reads were corrected and trimmed using Canu v. 1.5 (67). The long and short reads were used for genome assembly with Unicycler v. 0.4.8 in hybrid assembly mode (68). Genome assembly statistics were calculated using Quast v. 5.0.2 (69). As is common practice (70), short contigs (<500 bp) were removed from the assemblies using Seqkit v. 0.13.2 (71).

### Genome assembly of public data and sample set curation.

We complemented our set of novel genomes with publicly available data. To do so, we downloaded all of the available whole-genome assemblies of X. fastidiosa and *X. taiwanensis* (as an outgroup) from the National Center for Biotechnology Information (NCBI) and the Sequence Read Archive (SRA) databases on July 9, 2020 (Table S1). In addition, we downloaded the raw, short-read sequences for an additional 20 isolates (27, 42). For each isolate, we gathered information about its geographic origin and its host plant from NCBI and from the Pathosystems Resource Integration Center (PATRIC) database. To assemble the raw reads from the 20 unassembled accessions into genomes, we assessed quality, trimmed the reads, and applied SPAdes v. 3.14.0 (72) with the *–careful* option, following Vanhove et al. (2020) (Table S2) (27). If long reads were also available, as they were for 5 isolates from the work of Castillo et al. (2020) (42), then whole-genome assembly was performed with Unicycler v. 0.4.8 in hybrid assembly mode (68).

In total, we gathered and generated 148 *Xylella* genome assemblies. From this set, we removed isolates that did not have information about their host isolation source or were lab-derived recombinant strains. The remaining 129 genomes were reannotated by the same method, based on Prokka v. 1.14.6 analysis, which we applied to the new genomes to ensure homogeneity. The Prokka analyses were then input into Roary v. 3.13.0 with options *-i 80 -cd 100 -e -n -z* to obtain a core gene alignment for initial comparisons among isolates (35, 73). Here we defined core genes as those that were detectable in 100% of the samples. This core set was aligned with MAFFT and polished using gBlocks v. 0.91b (74–76). The polished alignment was used as an input for RAxML v. 8.2.12 to build a preliminary phylogenetic tree (77), which we used to evaluate and curate the isolates (Fig. S1).

To curate the data set, we created a distance matrix from the RAxML phylogenetic tree, using the Tree and Reticulogram Reconstruction (T-REX) server (78). Many of the genomes, most of which were gathered for population genomic analyses, were sampled from the same plant host and were nearly identical, genetically. To limit sampling biases in our species-wide study, we removed clones and near-clones based on the distance matrix. That is, if two or more isolates had a pairwise distance of ≤0.0001 and came from the same host, we retained the isolate with the more contiguous assembly. We also used CheckM (79) to assess genome completeness based on a set of conserved single copy genes (Table S3). After applying these filters, our final data set consisted of 63 X. fastidiosa genomes and one outgroup genome (*X. taiwanensis* PLS229) that were isolated from 23 distinct plant host species (Table S1).

### Pan-genome analysis.

To perform a pan-genome analysis, we applied Roary to the 64 *Xylella* genomes using the *gff* files from Prokka as an input. Roary was applied with the option *-i 80*, as used in previous microbial studies (45, 70), to lower the BLASTP sequence identity to 80% from the default 95%. We defined a core gene as a gene present in 95% of the isolates used in the analysis (i.e., a core gene was present in at least 60 of the 63 X. fastidiosa accessions). From the Roary output, we extracted a representative nucleotide sequence of each core and accessory gene using cdbfasta (https://github.com/gpertea/cdbfasta) and translated the nucleotide sequence to amino acids using the transeq command from EMBL-EBI (80). The representative sequences were the basis for functional categorization, using the eggNOG-mapper v. 2 (81, 82), of both the core and the accessory genes. GO enrichment analyses were performed online at (http://geneontology.org) using Xanthomonas campestris as the reference list (83). To explore function further, we also used the Conserved Domain Database online tool (https://www.ncbi.nlm.nih.gov/cdd/) to identify protein domains.

### Phylogenetic tree construction.

We used the core gene alignment from Roary to build a phylogenetic tree, based on a subset of genes that were present in all 63 X. fastidiosa samples and the *X. taiwenensis* outgroup. To do so, we curated the alignments with gBlocks v. 0.91b (74), used the polished alignment as an input for IQtree v. 2.0.3, and selected the best nucleotide model for phylogenetic tree construction (84, 85). We ultimately constructed an unrooted tree using the GTR+F+R8 model with RAxML (Stamatakis 2014) (77), using the “best tree” option. The phylogenetic trees were visualized and annotated using the ape package v. 5.5 in R v. 4.0.2 (86, 87). We used the most likely phylogeny to test for associations between phylogenetic relatedness, geography, and host isolation source (plant taxonomic order information taken from https://www.itis.gov/) via ANOSIM implemented in the vegan package v. 2.5-7 in R (88).

X. fastidiosa is naturally transformant and undergoes homologous recombination (9, 89), but recombined genomic regions can obscure vertical phylogenetic relationships. To account for potential recombination among the X. fastidiosa genomes, we applied Gubbins v. 3.2.1 (36, 90), again using the subset of genes that were found in all 64 samples. From this input, Gubbins identified regions that were likely to have undergone recombination and removed them from the alignment. We then built a phylogeny from this recombination-adjusted core gene alignment using RAxML, as described above. We assessed the congruence between the two phylogenetic trees (i.e., with and without the removal of potentially recombining regions) using phytools v. 1.0-1 in R (91).

Finally, we also built a neighbor-joining (NJ) tree based on the presence-absence matrix of accessory genes. We first calculated the Euclidean distances from the presence-absence matrix of the accessory genes using the *dist* function in R (92). We then built an NJ tree from the Euclidean distances using the ape package in R (86). We also utilized the ANOSIM and Mantel test (in the vegan package) to measure the correlation between accessory gene content and phylogenetic relatedness. The Mantel test required two distance matrices, which were the Euclidean distances estimated from the accessory gene presence-absence matrix and the distances from the RAxML core gene phylogeny generated by the Tree and Reticulogram Reconstruction (T-REX) server (78).

### Gain and loss of accessory genes.

We utilized GLOOME to investigate gene gain and loss dynamics along the core phylogenetic tree of X. fastidiosa (93). GLOOME uses a mixture-model approach coupled with maximum-likelihood inference to infer rates of gains and loss of genes along the branches of a phylogeny. It takes as inputs the phylogenetic topology (in this case, the phylogenetic topology based on the core genes) and a presence-absence matrix of genes. The pattern of genetic presence and absence was obtained through M1CR0B1AL1Z3R, as recommended by the GLOOME authors, and then directly input into GLOOME, using the default settings (94). The default settings included a fixed rate of gene gains and losses with gamma distributed rates across genes (or sites). Among the outputs, GLOOME returned two phylogenetic trees with branch lengths representing either the number of expected gain events or the number of loss events on each branch. As recommended (93), branch lengths representing relative gain and loss rates were extracted from the phylogenetic trees using FigTree v. 1.4.4 (http://tree.bio.ed.ac.uk/software/figtree/). To normalize the expected gain (or loss) events with the sequence divergence, we calculated the ratio of inferred gain (or loss) against the branch lengths of the sequence-based core phylogeny. Outlier branches with excess normalized gains or losses were identified using the interquartile range criterion.

### Positive selection analyses.

We employed *codeml* from PAML v. 4.9 to calculate *ω*, the ratio of nonsynonymous to synonymous rates (95, 96). We performed a *codeml* analysis on the nucleotide alignments of the single-copy core genes, single-copy accessory genes, and multicopy genes (defined as genes with two or more copies in a single accession). For all tests, we required at least four sequences, the minimum number suggested for *codeml* analysis (http://abacus.gene.ucl.ac.uk/software/pamlFAQs.pdf). For each gene and sequence set, we ran analyses by generating an unrooted maximum-likelihood tree for each gene based on the DNA alignment, using RAxML v. 8.2.12. This approach recognizes that the phylogeny of a single gene may not follow the consensus phylogeny due to a history of recombination. For completeness, however, we also performed *codeml* analyses by assuming the global phylogeny for the subset of genes that were present in all 64 samples. The outcomes of the two approaches were highly correlated (Fig. S2), and so, for simplicity, we focused on results based on phylogenies inferred separately for each gene.

Given the input phylogenies, we performed *codeml* analyses that relied on calculating likelihood ratios (LRs) under various models (96). Briefly, we used the models to test the null hypothesis that *ω* = 1.0 against the alternative of positive selection (*ω* > 1.0) in two different ways. The first was a global test across the entirely phylogeny of a gene (i.e., across all branches and all sites). This test requires the comparison of two models: one (Model = 0 with Fix_omega = 1 and Omega = 1 in the *codeml* control file) that estimates a single *ω* from the data and another that sets *ω* = 1.0 (Model = 0 with Fix_omega = 0 in the *codeml* control file). The two models yielded evidence for positive selection when the initial *ω* estimate was >1.0 and when the likelihoods of the two models differed significantly, based on *P* < 0.01 after FDR correction. The second set of analyses was across sites (i.e., testing for genes with variable selection pressure across sites). For each gene, we first compared models M0 and M3 to test for heterogeneity in evolutionary rates across codons. If that test was significant, we then compared sites models M1a and M2a from *codeml* to test for specific codons with evidence of positive selection (*ω* > 1.0). For all of the summary statistics of *ω*, we excluded estimates of *ω* that were greater than 10 as potentially unreliable due to either a low d_s_ or a poorly resolved optimization. Individual codon residues under positive selection were identified using the empirical Bayes analysis in *codeml*.

### Data availability.

All high-throughput sequence data generated in this study have been submitted to the NCBI Sequence Read Archive database at https://www.ncbi.nlm.nih.gov/sra and can be accessed with project number PRJNA833428.
