# Supplementary material for: Using Genomes and Evolutionary Analyses to Screen for Host-Specificity and Positive Selection in the Plant Pathogen Xylella fastidiosa
Source: Appl Environ Microbiol. 2022 Sep 12;88(18):e01220-22. doi: 10.1128/aem.01220-22 (PMC9499020; doi:10.1128/aem.01220-22)
Supplement: Supplemental file 1 — Tables S5 to S8 and Fig. S1 to S4. Download aem.01220-22-s0001.pdf, PDF file, 1.5 MB [file aem.01220-22-s0001.pdf]

Supplementary Table 5: GO analysis of core genes

GO biological process complete	REFLIST	upload	expected	over/under	fold Enrichment	P-value
pyrimidine-containing compound biosynthetic process (GO:0072528)	20	16	2.99	+	5.35	1.91E-03
translation (GO:0006412)	105	73	15.71	+	4.65	8.14E-19
aromatic amino acid family biosynthetic process (GO:0009073)	22	15	3.29	+	4.56	1.51E-02
regulation of developmental process (GO:0050793)	28	19	4.19	+	4.54	1.32E-03
peptide biosynthetic process (GO:0043043)	113	76	16.91	+	4.5	4.72E-19
regulation of cell shape (GO:0008360)	27	18	4.04	+	4.46	2.93E-03
regulation of cell morphogenesis (GO:0022604)	27	18	4.04	+	4.46	2.93E-03
regulation of anatomical structure morphogenesis (GO:0022603)	27	18	4.04	+	4.46	2.93E-03
ribonucleoside monophosphate metabolic process (GO:0009161)	29	19	4.34	+	4.38	1.93E-03
amide biosynthetic process (GO:0043604)	140	91	20.95	+	4.34	1.53E-22
nucleoside monophosphate biosynthetic process (GO:0009124)	31	20	4.64	+	4.31	1.26E-03
peptide metabolic process (GO:0006518)	133	85	19.9	+	4.27	1.63E-20
nucleoside monophosphate metabolic process (GO:0009123)	36	23	5.39	+	4.27	2.46E-04
RNA methylation (GO:0001510)	22	14	3.29	+	4.25	4.84E-02
ribonucleoside monophosphate biosynthetic process (GO:0009156)	27	17	4.04	+	4.21	9.32E-03
pyrimidine-containing compound metabolic process (GO:0072527)	27	17	4.04	+	4.21	9.32E-03
cell cycle (GO:0007049)	42	26	6.28	+	4.14	6.80E-05
cellular amide metabolic process (GO:0043603)	178	104	26.63	+	3.91	2.08E-23
ribonucleotide biosynthetic process (GO:0009260)	52	30	7.78	+	3.86	2.33E-05
ribose phosphate biosynthetic process (GO:0046390)	53	30	7.93	+	3.78	3.21E-05
ribonucleotide metabolic process (GO:0009259)	78	44	11.67	+	3.77	2.47E-08
glycosaminoglycan biosynthetic process (GO:0006024)	32	18	4.79	+	3.76	1.64E-02
aminoglycan biosynthetic process (GO:0006023)	32	18	4.79	+	3.76	1.64E-02
peptidoglycan biosynthetic process (GO:0009252)	32	18	4.79	+	3.76	1.64E-02
ribosome biogenesis (GO:0042254)	47	26	7.03	+	3.7	3.50E-04
ribonucleoprotein complex biogenesis (GO:0022613)	47	26	7.03	+	3.7	3.50E-04
ribose phosphate metabolic process (GO:0019693)	82	45	12.27	+	3.67	2.94E-08
purine ribonucleotide biosynthetic process (GO:0009152)	42	23	6.28	+	3.66	1.79E-03

monocarboxylic acid biosynthetic process (GO:0072330)	42	23	6.28	+	3.66	1.79E-03
cellular component macromolecule biosynthetic process (GO:0070589)	33	18	4.94	+	3.65	2.23E-02
purine ribonucleotide metabolic process (GO:0009150)	66	36	9.87	+	3.65	3.05E-06
cell wall macromolecule biosynthetic process (GO:0044038)	33	18	4.94	+	3.65	2.23E-02
cellular macromolecule biosynthetic process (GO:0034645)	178	97	26.63	+	3.64	6.22E-20
gene expression (GO:0010467)	244	132	36.5	+	3.62	3.41E-28
tRNA metabolic process (GO:0006399)	65	35	9.72	+	3.6	6.45E-06
organonitrogen compound biosynthetic process (GO:1901566)	406	218	60.74	+	3.59	4.74E-51
sulfur compound biosynthetic process (GO:0044272)	56	30	8.38	+	3.58	8.05E-05
nucleotide biosynthetic process (GO:0009165)	71	38	10.62	+	3.58	1.69E-06
peptidoglycan-based cell wall biogenesis (GO:0009273)	36	19	5.39	+	3.53	1.92E-02
nucleoside phosphate biosynthetic process (GO:1901293)	72	38	10.77	+	3.53	2.27E-06
tRNA processing (GO:0008033)	40	21	5.98	+	3.51	7.90E-03
purine nucleotide biosynthetic process (GO:0006164)	44	23	6.58	+	3.49	3.25E-03
vitamin biosynthetic process (GO:0009110)	46	24	6.88	+	3.49	2.08E-03
water-soluble vitamin biosynthetic process (GO:0042364)	46	24	6.88	+	3.49	2.08E-03
purine nucleotide metabolic process (GO:0006163)	69	36	10.32	+	3.49	7.43E-06
cellular amino acid biosynthetic process (GO:0008652)	100	52	14.96	+	3.48	4.04E-09
cell division (GO:0051301)	54	28	8.08	+	3.47	3.51E-04
cellular nitrogen compound biosynthetic process (GO:0044271)	365	189	54.61	+	3.46	5.09E-41
cell wall biogenesis (GO:0042546)	37	19	5.54	+	3.43	2.56E-02
phospholipid biosynthetic process (GO:0008654)	45	23	6.73	+	3.42	4.32E-03
phospholipid metabolic process (GO:0006644)	45	23	6.73	+	3.42	4.32E-03
organophosphate biosynthetic process (GO:0090407)	135	69	20.2	+	3.42	2.08E-12
purine-containing compound biosynthetic process (GO:0072522)	47	24	7.03	+	3.41	2.77E-03
water-soluble vitamin metabolic process (GO:0006767)	49	25	7.33	+	3.41	1.77E-03
vitamin metabolic process (GO:0006766)	49	25	7.33	+	3.41	1.77E-03
ncRNA metabolic process (GO:0034660)	98	50	14.66	+	3.41	1.78E-08
alpha-amino acid biosynthetic process (GO:1901607)	85	43	12.72	+	3.38	5.63E-07

carboxylic acid biosynthetic process (GO:0046394)	157	79	23.49	+	3.36	3.26E-14
organic acid biosynthetic process (GO:0016053)	159	79	23.79	+	3.32	5.71E-14
RNA processing (GO:0006396)	77	38	11.52	+	3.3	9.26E-06
ncRNA processing (GO:0034470)	73	36	10.92	+	3.3	2.27E-05
external encapsulating structure organization (GO:0045229)	41	20	6.13	+	3.26	2.84E-02
nucleotide metabolic process (GO:0009117)	107	52	16.01	+	3.25	2.83E-08
small molecule biosynthetic process (GO:0044283)	225	109	33.66	+	3.24	1.31E-19
sulfur compound metabolic process (GO:0006790)	93	45	13.91	+	3.23	6.77E-07
carbohydrate derivative biosynthetic process (GO:1901137)	152	73	22.74	+	3.21	3.79E-12
dicarboxylic acid metabolic process (GO:0043648)	50	24	7.48	+	3.21	6.23E-03
nucleoside phosphate metabolic process (GO:0006753)	111	53	16.61	+	3.19	3.04E-08
purine-containing compound metabolic process (GO:0072521)	78	37	11.67	+	3.17	3.20E-05
cellular biosynthetic process (GO:0044249)	616	292	92.16	+	3.17	1.39E-64
organic substance biosynthetic process (GO:1901576)	625	294	93.51	+	3.14	1.42E-64
organophosphate metabolic process (GO:0019637)	185	87	27.68	+	3.14	2.42E-14
macromolecule biosynthetic process (GO:0009059)	251	118	37.55	+	3.14	1.49E-20
aromatic compound biosynthetic process (GO:0019438)	235	109	35.16	+	3.1	2.01E-18
organic cyclic compound biosynthetic process (GO:1901362)	266	122	39.8	+	3.07	1.01E-20
biosynthetic process (GO:0009058)	650	296	97.25	+	3.04	1.50E-62
heterocycle biosynthetic process (GO:0018130)	251	113	37.55	+	3.01	2.33E-18
RNA modification (GO:0009451)	56	25	8.38	+	2.98	1.74E-02
RNA metabolic process (GO:0016070)	170	75	25.43	+	2.95	6.31E-11
nucleobase-containing small molecule metabolic process (GO:0055086)	147	64	21.99	+	2.91	7.92E-09
cellular component biogenesis (GO:0044085)	148	64	22.14	+	2.89	9.35E-09
DNA repair (GO:0006281)	72	31	10.77	+	2.88	2.25E-03
nucleobase-containing compound biosynthetic process (GO:0034654)	165	71	24.69	+	2.88	6.94E-10
nucleic acid phosphodiester bond hydrolysis (GO:0090305)	56	24	8.38	+	2.86	3.43E-02
regulation of biological quality (GO:0065008)	73	31	10.92	+	2.84	2.71E-03
carbohydrate derivative metabolic process (GO:1901135)	231	98	34.56	+	2.84	3.48E-14
lipid biosynthetic process (GO:0008610)	95	40	14.21	+	2.81	1.80E-04
cellular amino acid metabolic process (GO:0006520)	190	80	28.43	+	2.81	5.80E-11

cellular nitrogen compound metabolic process (GO:0034641)	703	295	105.18	+	2.8	1.13E-55
alpha-amino acid metabolic process (GO:1901605)	138	57	20.65	+	2.76	6.99E-07
cellular component organization or biogenesis (GO:0071840)	197	81	29.47	+	2.75	1.33E-10
carboxylic acid metabolic process (GO:0019752)	327	134	48.92	+	2.74	2.14E-19
oxoacid metabolic process (GO:0043436)	334	136	49.97	+	2.72	1.32E-19
small molecule metabolic process (GO:0044281)	507	203	75.85	+	2.68	2.22E-31
cellular response to DNA damage stimulus (GO:0006974)	80	32	11.97	+	2.67	6.09E-03
organic acid metabolic process (GO:0006082)	341	136	51.02	+	2.67	6.21E-19
cellular component organization (GO:0016043)	152	60	22.74	+	2.64	9.23E-07
monocarboxylic acid metabolic process (GO:0032787)	123	48	18.4	+	2.61	5.03E-05
cellular response to stress (GO:0033554)	107	41	16.01	+	2.56	7.27E-04
organic cyclic compound metabolic process (GO:1901360)	614	233	91.86	+	2.54	2.41E-34
heterocycle metabolic process (GO:0046483)	589	223	88.12	+	2.53	3.20E-32
cellular aromatic compound metabolic process (GO:0006725)	587	220	87.82	+	2.51	4.84E-31
cellular lipid metabolic process (GO:0044255)	127	47	19	+	2.47	2.53E-04
cellular protein metabolic process (GO:0044267)	255	93	38.15	+	2.44	5.67E-10
nucleobase-containing compound metabolic process (GO:0006139)	495	179	74.06	+	2.42	2.37E-22
organonitrogen compound metabolic process (GO:1901564)	781	280	116.85	+	2.4	4.72E-40
cellular metabolic process (GO:0044237)	1323	455	197.93	+	2.3	1.26E-83
nitrogen compound metabolic process (GO:0006807)	1124	384	168.16	+	2.28	2.39E-60
response to stress (GO:0006950)	143	48	21.39	+	2.24	2.57E-03
nucleic acid metabolic process (GO:0090304)	354	118	52.96	+	2.23	3.85E-11
phosphorus metabolic process (GO:0006793)	328	107	49.07	+	2.18	2.48E-09
cellular macromolecule metabolic process (GO:0044260)	567	184	84.83	+	2.17	1.59E-18
phosphate-containing compound metabolic process (GO:0006796)	320	103	47.88	+	2.15	1.26E-08
lipid metabolic process (GO:0006629)	150	48	22.44	+	2.14	6.63E-03
organic substance metabolic process (GO:0071704)	1435	449	214.69	+	2.09	7.90E-69
primary metabolic process (GO:0044238)	1277	394	191.05	+	2.06	6.86E-52
protein metabolic process (GO:0019538)	383	116	57.3	+	2.02	1.81E-08
metabolic process (GO:0008152)	1590	477	237.88	+	2.01	3.77E-72
macromolecule metabolic process (GO:0043170)	821	239	122.83	+	1.95	2.28E-20
cellular process (GO:0009987)	1918	543	286.95	+	1.89	2.91E-90
biological_process (GO:0008150)	2336	582	349.49	+	1.67	3.82E-88
Unclassified (UNCLASSIFIED)	1768	32	264.51	-	0.12	0.00E+00

Supplementary Table 6: GO analysis of accessory genes

GO biological process complete	REFLIST	upload	expected	over/under	fold Enrichment	P-value
glutamine family amino acid biosynthetic process (GO:0009084)	17	8	0.94	+	8.51	1.46E-02
glutamine metabolic process (GO:0006541)	17	8	0.94	+	8.51	1.46E-02
tRNA aminoacylation for protein translation (GO:0006418)	25	10	1.38	+	7.23	4.31E-03
tRNA aminoacylation (GO:0043039)	25	10	1.38	+	7.23	4.31E-03
amino acid activation (GO:0043038)	26	10	1.44	+	6.95	5.69E-03
chromosome organization (GO:0051276)	27	9	1.49	+	6.03	4.03E-02
glutamine family amino acid metabolic process (GO:0009064)	37	11	2.05	+	5.37	1.51E-02
purine nucleotide biosynthetic process (GO:0006164)	44	12	2.43	+	4.93	1.34E-02
tRNA metabolic process (GO:0006399)	65	17	3.6	+	4.73	4.18E-04
purine-containing compound biosynthetic process (GO:0072522)	47	12	2.6	+	4.62	2.33E-02
ribose phosphate biosynthetic process (GO:0046390)	53	13	2.93	+	4.43	1.59E-02
ncRNA metabolic process (GO:0034660)	98	24	5.42	+	4.43	5.69E-06
nucleotide biosynthetic process (GO:0009165)	71	15	3.93	+	3.82	1.76E-02
cellular amino acid biosynthetic process (GO:0008652)	100	21	5.53	+	3.8	4.68E-04
translation (GO:0006412)	105	22	5.81	+	3.79	2.61E-04
nucleoside phosphate biosynthetic process (GO:1901293)	72	15	3.98	+	3.77	2.03E-02
ncRNA processing (GO:0034470)	73	15	4.04	+	3.71	2.34E-02
alpha-amino acid biosynthetic process (GO:1901607)	85	17	4.7	+	3.62	9.63E-03
RNA processing (GO:0006396)	77	15	4.26	+	3.52	4.00E-02
peptide biosynthetic process (GO:0043043)	113	22	6.25	+	3.52	7.74E-04
amide biosynthetic process (GO:0043604)	140	27	7.74	+	3.49	5.23E-05
cellular amino acid metabolic process (GO:0006520)	190	35	10.51	+	3.33	1.49E-06
RNA metabolic process (GO:0016070)	170	31	9.4	+	3.3	1.67E-05
peptide metabolic process (GO:0006518)	133	23	7.36	+	3.13	2.63E-03

organonitrogen compound biosynthetic process (GO:1901566)	406	68	22.46	+	3.03	3.08E-13
carboxylic acid biosynthetic process (GO:0046394)	157	26	8.68	+	2.99	1.23E-03
organic acid biosynthetic process (GO:0016053)	159	26	8.79	+	2.96	1.52E-03
gene expression (GO:0010467)	244	39	13.5	+	2.89	5.90E-06
cellular amide metabolic process (GO:0043603)	178	28	9.85	+	2.84	1.20E-03
alpha-amino acid metabolic process (GO:1901605)	138	21	7.63	+	2.75	3.83E-02
cellular macromolecule biosynthetic process (GO:0034645)	178	27	9.85	+	2.74	3.44E-03
small molecule biosynthetic process (GO:0044283)	225	34	12.45	+	2.73	1.99E-04
carboxylic acid metabolic process (GO:0019752)	327	48	18.09	+	2.65	1.45E-06
oxoacid metabolic process (GO:0043436)	334	49	18.47	+	2.65	9.46E-07
cellular nitrogen compound biosynthetic process (GO:0044271)	365	53	20.19	+	2.63	2.03E-07
organic acid metabolic process (GO:0006082)	341	49	18.86	+	2.6	1.49E-06
small molecule metabolic process (GO:0044281)	507	70	28.04	+	2.5	7.71E-10
nucleic acid metabolic process (GO:0090304)	354	47	19.58	+	2.4	3.16E-05
cellular component organization or biogenesis (GO:0071840)	197	26	10.9	+	2.39	4.79E-02
cellular biosynthetic process (GO:0044249)	616	81	34.07	+	2.38	7.21E-11
macromolecule biosynthetic process (GO:0009059)	251	33	13.88	+	2.38	6.22E-03
biosynthetic process (GO:0009058)	650	85	35.95	+	2.36	1.44E-11
organic substance biosynthetic process (GO:1901576)	625	81	34.57	+	2.34	1.28E-10
nucleobase-containing compound metabolic process (GO:0006139)	495	64	27.38	+	2.34	1.73E-07
cellular aromatic compound metabolic process (GO:0006725)	587	74	32.47	+	2.28	9.26E-09
heterocycle metabolic process (GO:0046483)	589	74	32.58	+	2.27	1.04E-08
cellular protein metabolic process (GO:0044267)	255	32	14.1	+	2.27	2.65E-02
organic cyclic compound metabolic process (GO:1901360)	614	77	33.96	+	2.27	3.34E-09

cellular nitrogen compound metabolic process (GO:0034641)	703	86	38.88	+	2.21	3.79E-10
organonitrogen compound metabolic process (GO:1901564)	781	94	43.2	+	2.18	3.34E-11
nitrogen compound metabolic process (GO:0006807)	1124	129	62.17	+	2.07	1.17E-16
cellular macromolecule metabolic process (GO:0044260)	567	64	31.36	+	2.04	3.36E-05
cellular metabolic process (GO:0044237)	1323	144	73.18	+	1.97	7.22E-18
protein metabolic process (GO:0019538)	383	41	21.18	+	1.94	4.45E-02
macromolecule metabolic process (GO:0043170)	821	87	45.41	+	1.92	4.67E-07
primary metabolic process (GO:0044238)	1277	133	70.63	+	1.88	7.50E-14
organic substance metabolic process (GO:0071704)	1435	148	79.37	+	1.86	1.76E-16
metabolic process (GO:0008152)	1590	158	87.95	+	1.8	5.66E-17
cellular process (GO:0009987)	1918	185	106.09	+	1.74	5.42E-23
biological_process (GO:0008150)	2336	198	129.21	+	1.53	3.01E-19
Unclassified (UNCLASSIFIED)	1768	29	97.79	-	0.3	0.00E+00

Supplementary Table 7: List of eight genes with evidence of positive selection based on the global test

**Gene**	**(M0)**[1]	**Likelihood ratio test P-value**[2]
cya	1.24241	0.00669718
group_454	2.06375	0.00918337
group_1057	2.2049	0.0029329
group_3049	2.49761	0.00248336
group_3542	1.94168	0.00235286
group_3757	6.53607	0.00434296
group_5674	1.58194	0.0079647
group_7848	9.60069	0.00868936

[1] M0 estimates a single w across the entire phylogeny of sequences

[2] The p-value of tests after FDR correction. LRT between the global model which estimates omega for each gene and the model which sets omega to 1 for each gene.

Supplementary Table 8: GO analysis of accessory genes with significant evidence for positive selection under the sites models M2a vs. M1a

GO biological process complete	REFLIST	upload	expected	over/under	fold Enrichment	P-value
protein secretion by the type IV secretion system (GO:0030255)	4	4	0.01	+	> 100	2.42E-06
secretion by the type IV secretion system (GO:0044097)	5	4	0.02	+	> 100	4.35E-06
protein transmembrane transport (GO:0071806)	40	4	0.13	+	31.57	4.41E-03
establishment of protein localization to extracellular region (GO:0035592)	48	4	0.15	+	26.31	8.66E-03
protein secretion (GO:0009306)	48	4	0.15	+	26.31	8.66E-03
protein localization to extracellular region (GO:0071692)	48	4	0.15	+	26.31	8.66E-03
secretion by cell (GO:0032940)	49	4	0.16	+	25.77	9.35E-03
secretion (GO:0046903)	49	4	0.16	+	25.77	9.35E-03
export from cell (GO:0140352)	51	4	0.16	+	24.76	1.09E-02
protein transport (GO:0015031)	84	5	0.27	+	18.79	2.76E-03
protein localization (GO:0008104)	88	5	0.28	+	17.94	3.43E-03
establishment of protein localization (GO:0045184)	88	5	0.28	+	17.94	3.43E-03
macromolecule localization (GO:0033036)	106	5	0.34	+	14.89	8.21E-03
nitrogen compound transport (GO:0071705)	129	6	0.41	+	14.68	9.20E-04
organic substance transport (GO:0071702)	168	6	0.53	+	11.27	4.08E-03
Unclassified (UNCLASSIFIED)	1768	3	5.6	-	0.54	0.00E+00

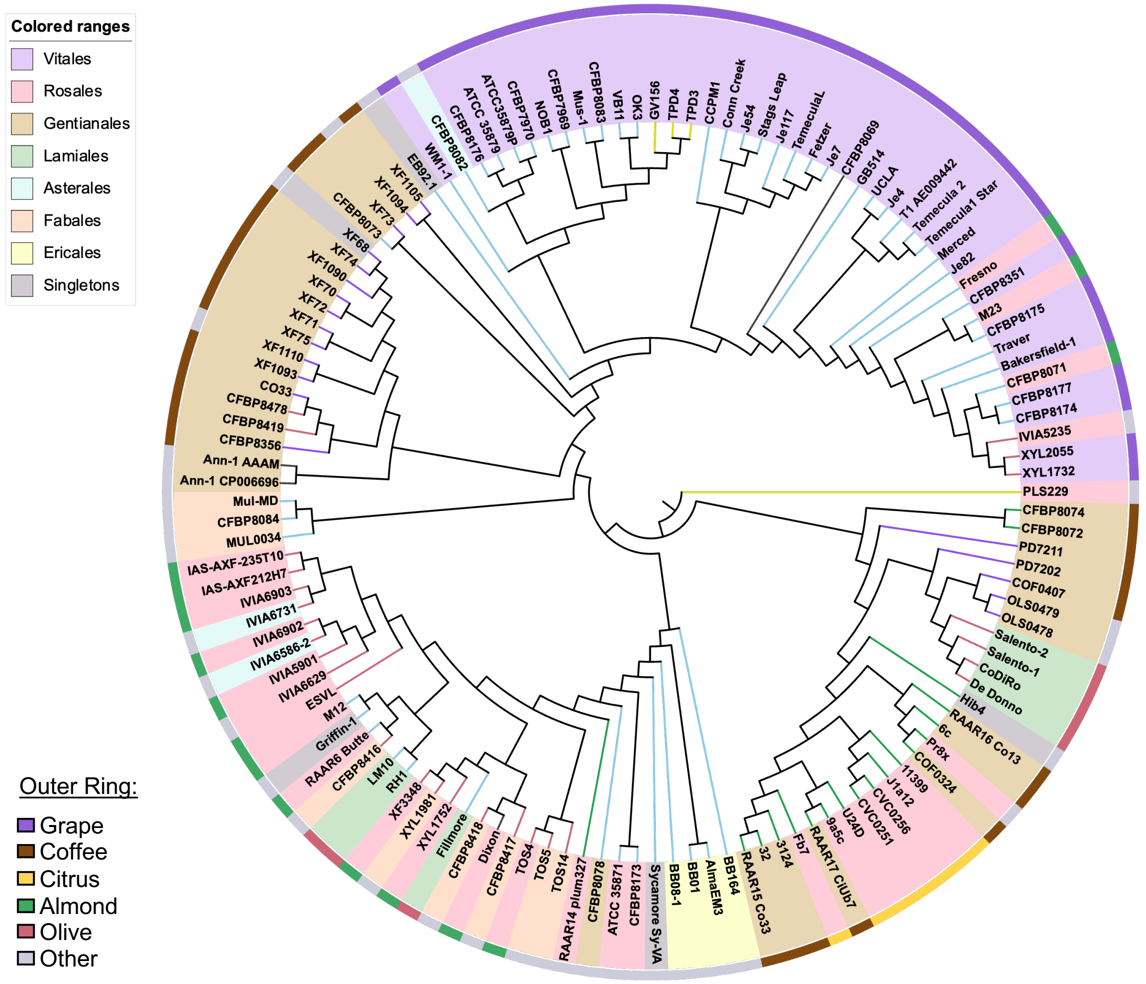

Supplementary Figure 1: Phylogenetic relationships of the set of 129 publicly available and novel *Xylella fastidiosa* and *X. taiwanensis* genomes gathered to develop this study. The outer ring denotes the specific plant host from which the bacteria was isolated, and the shaded ranges denote the host plant's taxonomic order. The branches are colored to denote the continent of isolation: North America (Blue), Central America (Purple), South America (Green), Europe (Red), Asia (Gold).

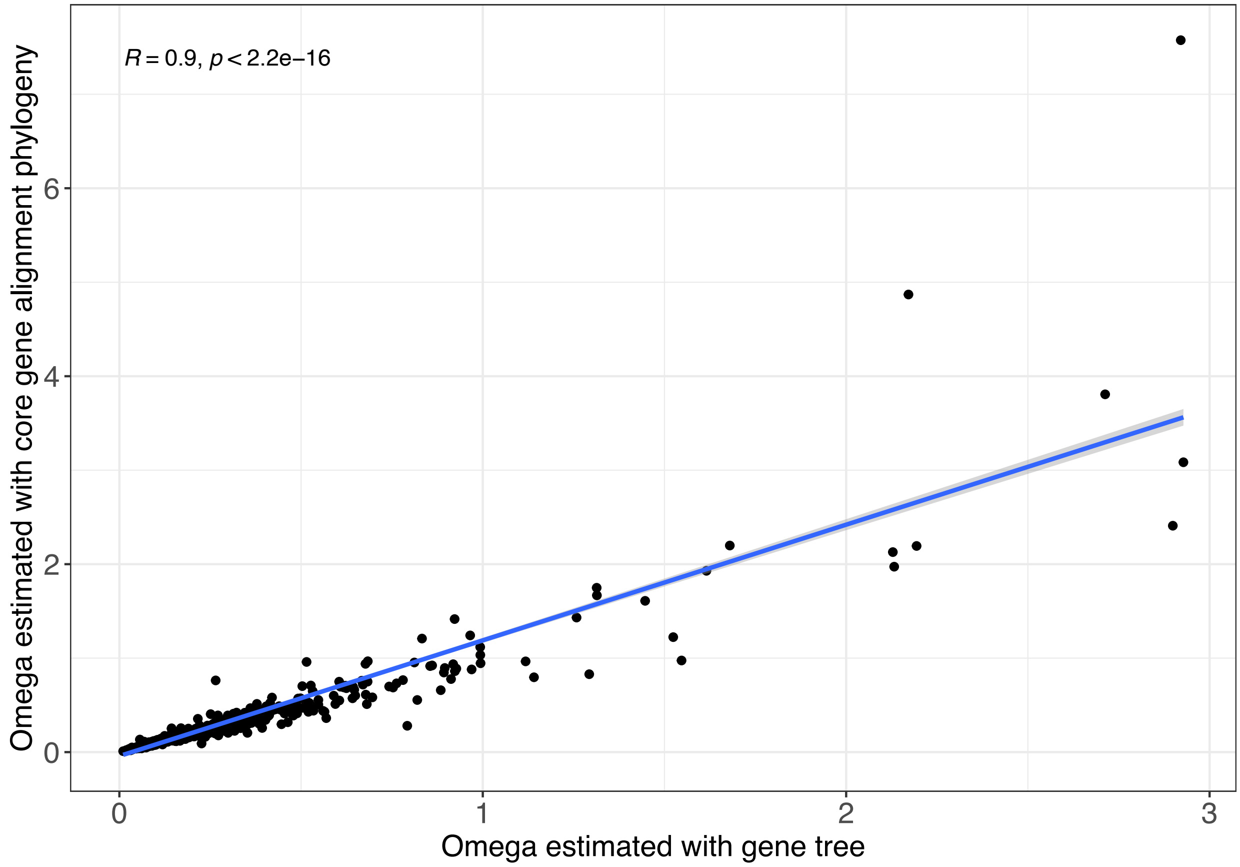

Supplementary Figure 2: Correlation plot between the values of omega estimated with two different methods: building an unrooted maximum-likelihood gene tree plotted on the x-axis and with the global phylogeny built from the core gene alignment on the y-axis.

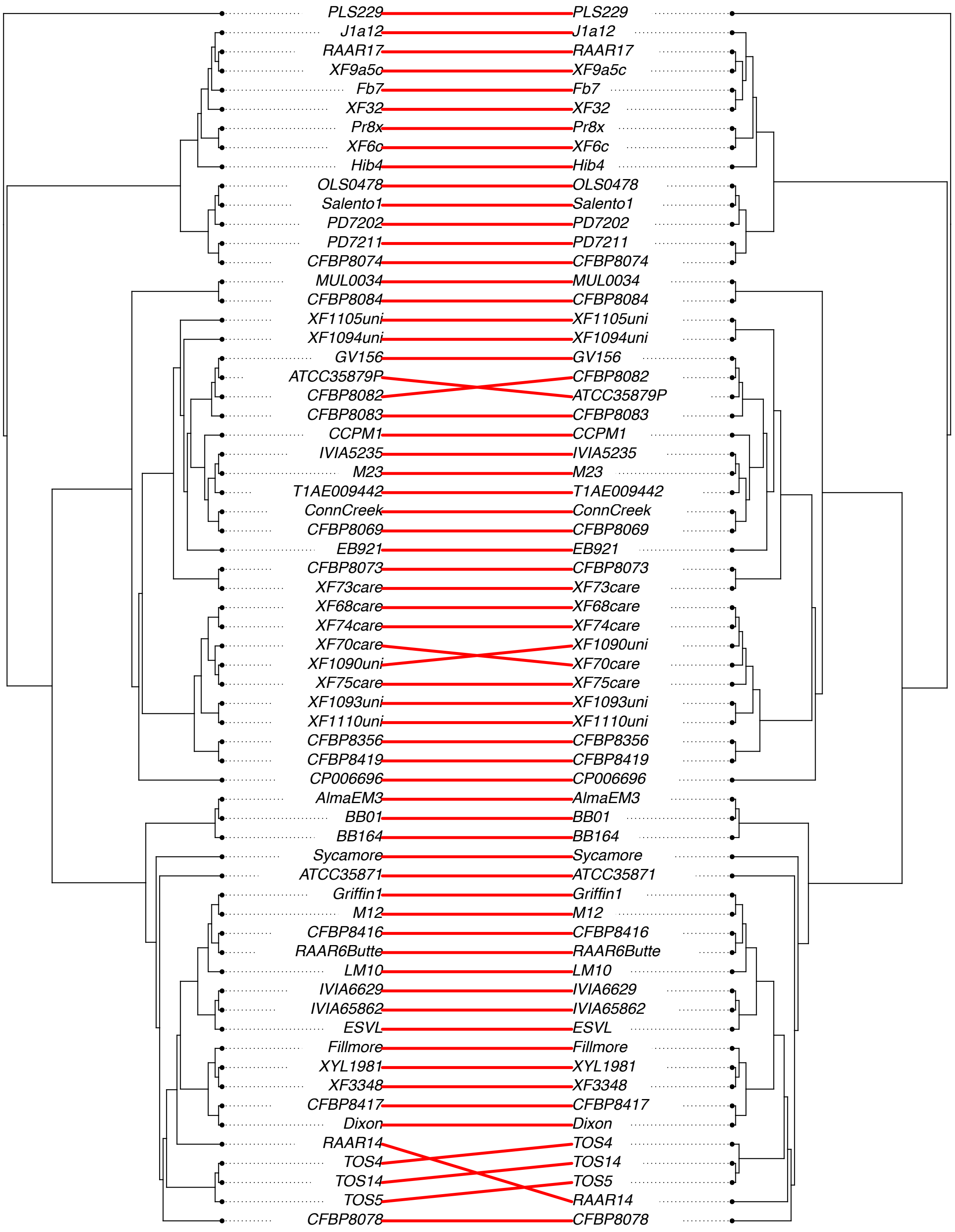

Supplementary Figure 3: Comparison of Gubbins recombination-corrected phylogeny (left) to the phylogeny built from the entire core gene alignment (right).

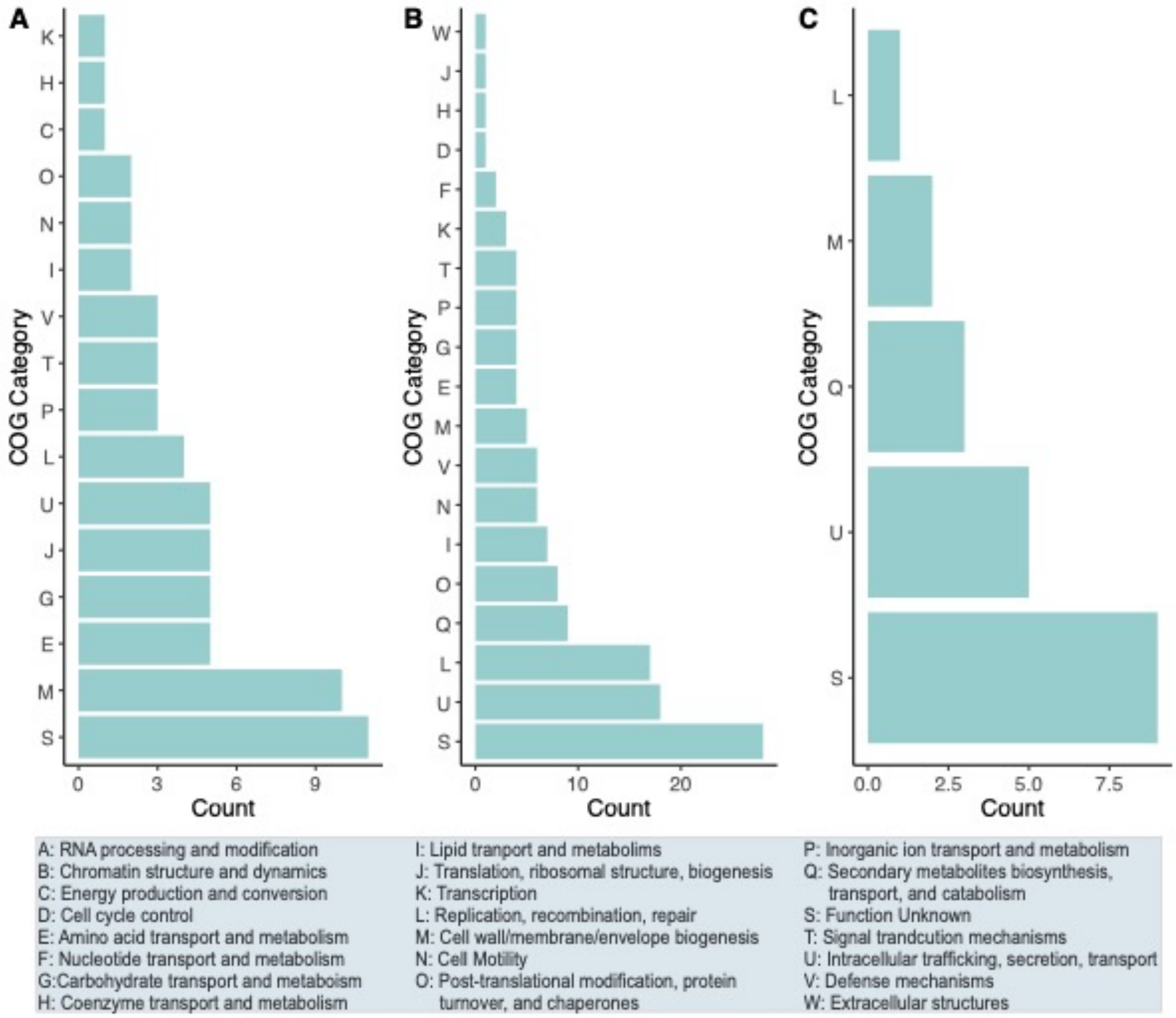

Supplementary Figure 4: The distribution of functional categories for A) the 67 core genes, B) the 201 accessory genes, and C) 33 multicopy genes with evidence for positive selection under the sites models. A key to the COG categories is provided in the bottom panel.
